# Prosody Discrimination by Songbirds (*Padda oryzivora*)

**DOI:** 10.1371/journal.pone.0047446

**Published:** 2012-10-17

**Authors:** Nozomi Naoi, Shigeru Watanabe, Kikuo Maekawa, Junko Hibiya

**Affiliations:** 1 Department of Psychology, Keio University, Tokyo, Japan; 2 The National Institute for Japanese Language and Linguistics (NINJAL), Tokyo, Japan; 3 College of Liberal Arts, International Christian University, Tokyo, Japan; University of South Florida, United States of America

## Abstract

In human verbal communication, not only lexical information, but also paralinguistic information plays an important role in transmitting the speakers’ mental state. Paralinguistic information is conveyed mainly through acoustic features like pitch, rhythm, tempo and so on. These acoustic features are generally known as prosody. It is known that some species of birds can discriminate certain aspects of human speech. However, there have not been any studies on the discrimination of prosody in human language which convey different paralinguistic meanings by birds. In the present study, we have shown that the Java sparrow (*Padda oryzivora*) can discriminate different prosodic patterns of Japanese sentences. These birds could generalize prosodic discrimination to novel sentences, but could not generalize sentence discrimination to those with novel prosody. Moreover, unlike Japanese speakers, Java sparrows used the first part of the utterance as the discrimination cue.

## Introduction

When we talk with another person, it appears that the semantic content of the spoken words carry the primary message. However, it is not only lexical information, but also paralinguistic information that plays an important role in transmitting the speaker’s intention, mental attitude, and focus through auditory features. These features include pitch, loudness, rhythm, and tempo [1]–[6], and are generally known as prosody. Humans cannot control non-linguistic information caused by physical factors, such as gender, or age, but we can intentionally vary prosody to express negative feelings even when the spoken text is positive. For example, using the same linguistic information of “lovely weather outside,” we can express a positive feeling about fine weather, or we can protest about the rainy weather by merely varying prosody.

In addition, paralinguistic data can transmit information that facilitates the listener’s comprehension [7]–[11]. In many languages, interrogative intonation has a high final pitch rising end, whereas declarative intonation has a falling end [12]–[15]. Moreover, Japanese people tend to elicit exclamatory rather than declarative identification when there is an increase in the height and magnitude of utterance of the initial pitch rise and the magnitude of the utterance of the final pitch fall increases [16].

Comprehension of prosody starts earlier than that of lexical information in typical development [17], [18]. Human infants can acquire rhythmic units of their maternal language in the first year after birth. Prosodic cues also seem to signal some syntactic structures, such as word boundaries [19]–[23]. In addition, human infants display sensitivity to paralinguistic cues to emotions such as happy and angry [24], and to approval or disapproval [25], even before the acquisition of language.

Previous studies have suggested that speech perception ability is not unique to humans (e.g., chimpanzees [26]; macaques [27]; cotton-top tamarin monkeys [28]; chinchillas [29]; and rats [30], [31]). Nonhuman primates and mammalian species are not the only species whose vocal communication appears to be closely related to that of humans. The vocal communication of avian species also shares several features with human speech [32], [33]. First, both birdsong and human speech are learned. Second, vocal learning requires the perception of the sounds, the capacity to learn to produce sounds, and the ability to relate the two. Third, both humans and some songbirds have critical or sensitive periods for vocal learning. Fourth, social feedback facilitates vocal development in both species. Finally, the neural region for vocal communication is lateralized in one hemisphere in humans and in some songbirds.

Because of these similarities between human vocal communication and bird songs, a number of experiments have investigated how avian species perceive various properties of auditory stimuli (e.g., zebra finches [34], [35]; Java sparrows [36]–[38]; budgerigars [34], [39]–[41]; Japanese quails [42], [43]; European starlings [44], [45]; pigeons [46]). However, most studies on speech perception in birds have involved the discrimination of linguistic units of human speech, such as vowels, consonants, words, and sentences. Bird songs and paralinguistic information in humans are especially similar in that both are conveyed not by the segmental but the supra-segmental features of vocalizations. A limited number of studies have examined prosody discrimination in animals, and most of them focused on the discrimination of language, based on rhythmic cues in prosody [38], [47], [48].

However, there have been no studies on the discrimination by birds of prosody in human language, which is known to convey different paralinguistic meanings. Among prosodic features, mean pitch and pitch range in spoken language may be the most salient cues for transmitting the human speaker’s intention and affective states [49], [50].

This study investigated whether prosody in the Japanese language, which conveys different paralinguistic meanings, could be discriminated by Java sparrows (*Padda oryzivora*). Java sparrows are known to have the ability to discriminate between complex tonal stimuli: concord and discord [37], the music of Bach and that of Schoenberg [36], and stories uttered in English and Chinese [38]. If they can discriminate Japanese sentences with different prosody, it would suggest that the capacity to discriminate paralinguistic information in spoken language is not unique to humans and not specific for language processing and that remotely related species such as songbirds also have a mechanism that can be used to discriminate these prosodic stimuli.

In Experiment 1, five adult Java sparrows (*Padda oryzivora*) were trained to discriminate between two Japanese sentences with the same text but that were uttered with different prosody. They were then tested with a novel text to assess their generalization strategies. The birds were also tested with hybrid stimuli to examine which acoustical cues played a role in their discrimination process. In Experiment 2, we examined whether birds could discriminate different linguistic information that express the same paralinguistic meanings, and the discrimination generalized to the stimuli with different prosody. By comparing the results of Experiments 1 and 2, we clarified the discriminative properties of prosody and text in the songbird.

## Results

### Experiment 1: Prosody Discrimination

#### Training

All birds were able to learn the discrimination task. Three birds in an Admiration Positive Stimulus Group (Admiration Group) reached the criterion in sessions 17, 24, and 28. Two birds in a Suspicion Positive Stimulus Group (Suspicion Group) reached the criterion in sessions 24 and 27.

#### Test 1

**Figure 1:**
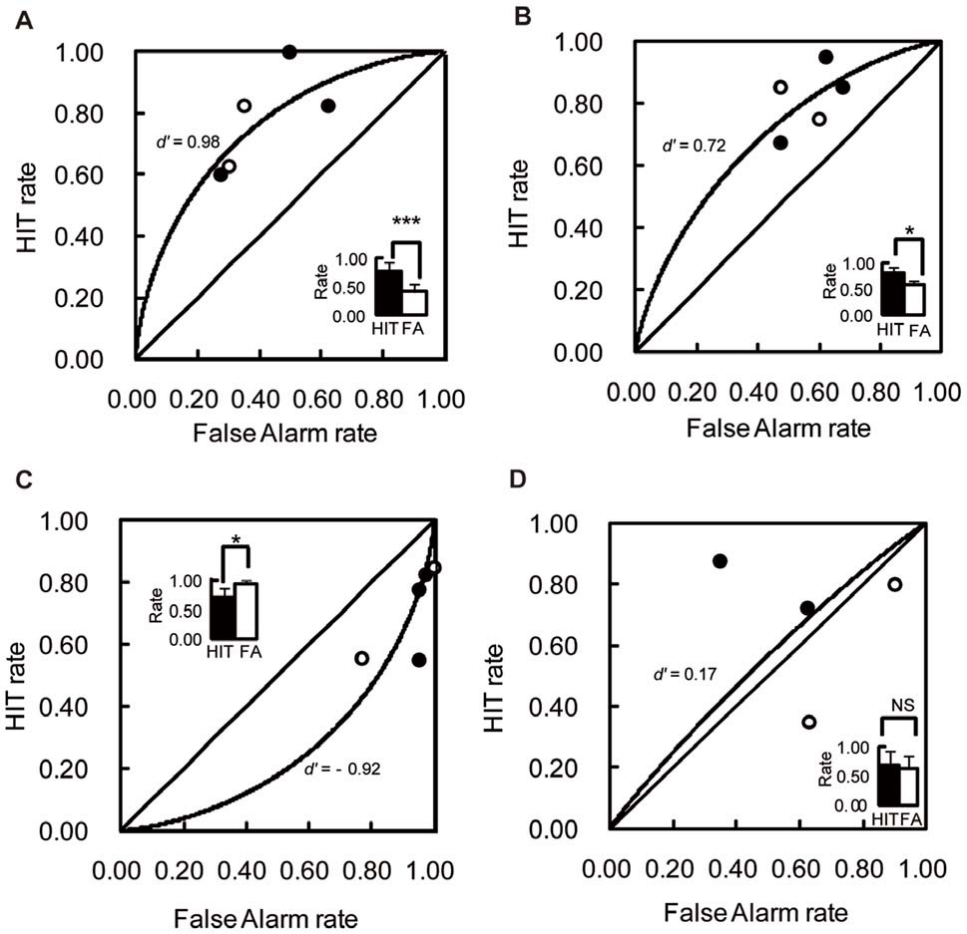
Results of generalization tests. The abscissa is the probability of a false alarm, and the ordinate is the probability of a hit. ROC (Relative Operating Characteristics) curve and the value of *d’* are depicted in the figure. The bar graph displays the means of hits and false alarms. * *p*<0.05, ** *p*<0.01, *** *p*<0.001 A. Results of the prosody generalization tests (Test 1) in Experiment 1. Open circles indicate birds in the Admiration Group, and closed circles, birds in the Suspicion Group. B. Results of the test with hybrid stimuli (Test 2-1) in Experiment 1. C. Results of the test with hybrid stimuli (Test 2-2) in Experiment 1. D. Results of the text generalization test in Experiment 2. Open circles indicate birds trained to respond to the text “so'H desu ka” and closed circles indicate birds trained to respond to the text “ana'ta desu ka” respectively. NS: non-significant.

Figure 1A shows the results of Test 1. Because there was no systematic difference between Admiration Group and Suspicion Group, the results of the two groups were combined for analysis. There was no significant difference between the mean correct response rates for the first and second replicates of Test 1 (paired t-test, *t* (4) = 1.04, *p* = 0.36). The mean rates of correct responses for each bird were 0.60, 0.73, 0.66, 0.74, and 0.66, respectively. There was a significant difference from the chance level (one group t-test, *t* (4) = 6.91, *p* = 0.002). A paired t-test revealed a significant difference between the probability of the correct perching (hit) and error perching (FA) (*t* (4) = 6.66, *p* = 0.003). Reaction time of perching also suggested the maintenance of prosodic discrimination during the test. The birds tended to respond more slowly in FA than in hit (paired t-test, *t* (4) = 2.39, *p* = 0.08). The value of discriminability index (*d’*) was based on a signal detection theory and had a value of 0.98.

#### Test 2

There was no significant difference between the mean correct response rates of the first and second replicates of Test 2-1 and Test 2-2 respectively (Test2-1; paired t-test, *t* (4) = 0.39, *p* = 0.72, Test 2-2; paired t-test, *t* (4) = 0.36, *p* = 0.74, respectively). The birds discriminated the hybrid stimuli correctly in Test 2-1 (Figure 1B). The mean rates of correct responses for each bird were 0.60, 0.66, 0.59, 0.58, and 0.69, respectively. The probability of hit had significant difference from that of the FA (paired t-test, *t* (4) = 5.56, *p* = 0.005) and the correct response rate also differed significantly from the chance level (*t* (4) = 5.74, *p* = 0.005). The value of *d’* was over 0.72. However, the birds preferred the incorrect hybrid stimulus in Test 2-2 (Figure 1C). The mean rates of correct responses for each bird were 0.43, 0.30, 0.41, 0.43, and 0.39, respectively. Probability comparison of hit, FA, and the correct response rate showed a significantly high response rate to the incorrect stimuli (*t* (4) = 4.65 and 4.47, *p* = 0.01 and 0.01 respectively). The value of *d’* was - 0.92. The birds responded to the correct hybrid faster than to the incorrect hybrid in Test 2-1 (*t* (4) = 9.83, *p*<0.001) and they showed the reversed tendency in Test 2-2 (*t* (4) = 10.30, *p*<0.001).

### Experiment 2: Sentence Discrimination

#### Training

All birds were able to learn the discrimination task. Two birds in a “so’H desu ka” Positive Stimulus Group (“so’H desu ka” Group) reached the criterion in sessions 26 and 37. Two birds in an “ana’ta desu ka” Positive Stimulus Group (“ana’ta desu ka” Group) reached the criterion in sessions 18 and 19. In comparison with the prosodic discrimination, the sentence discrimination was not hard for the sparrows to learn (two tailed t-test, *t* (7) = 0.27, *p* = 0.79).

#### Test

Figure 1D shows the results of the generalization test. There was no significant difference between the first and second test sessions (paired t-test, *t* (3) = 0.01, *p* = 0.99). The mean rates of correct responses for each bird were 0.77, 0.45, 0.55, and 0.36, respectively. The discrimination also did not differ from the chance level (one group t-test, *t* (3) = 0.37, *p* = 0.74). The probability of hit and FA did not differ as well (paired t-test, *t* (3) = 0.36, *p* = 0.74). The value of *d’* was 0.17.

## Discussion

The results of Experiment 1 demonstrated the generalization of prosodic discrimination to new stimulus sets. Songbirds are known to have better absolute pitch discrimination than humans [51]–[53]. For example, zebra finches (*Taeniopygia guttata*) could be trained to discriminate fine tones spaced 120 Hz apart, in the spectral region between 980 and 5660 Hz, parsed into eight ranges of five tones each [53]. Thus, the present results could potentially be explained by absolute pitch discrimination between training and test stimuli. However, as mentioned in the description of the stimuli, the beginning parts of the two training stimuli had very similar mean pitch values: the difference in mean pitch of the beginning parts of Stimuli A and S was only 4.5 Hz. In addition, the beginning parts of the two test stimuli did not differ greatly in pitch either (the difference in mean pitch of the beginning parts of Test Stimuli A and S was 43.0 Hz). Furthermore, the mean pitch of Stimulus A was lower than that of Test Stimulus A, and that of Stimulus S was higher than that of Test Stimulus S. Therefore, it is unlikely that the present results are due to discrimination of differences in absolute pitch between training and test speech samples. Thus, it could be concluded that the birds could identify a difference in prosody, regardless of the linguistic content.

Additionally, the results of Test 2, using hybrid stimuli, suggest that the birds used the beginning part of the phrase as a cue for their prosodic discrimination. In other words, the birds responded if the beginning parts consisted of the prosody associated with food in training. This tendency was so strong and consistent that regardless of the contingencies of reinforcement that were effective during the test, the birds consistently responded to incorrect stimuli in Test 2-2. This result seems consistent with a previous study [54] demonstrating that red-winged blackbirds and brown-headed cowbirds attend primarily to the introductory elements and disregard information in the final elements when identifying both alien and conspecific songs, whereas humans attend primarily to the final song elements. Future research could focus on testing whether Java sparrows completely disregard information in the final elements, by presenting birds with an “inverted hybrid stimulus,” in which the second half of a sentence is placed at the beginning, and the first half at the end.

**Figure 2:**
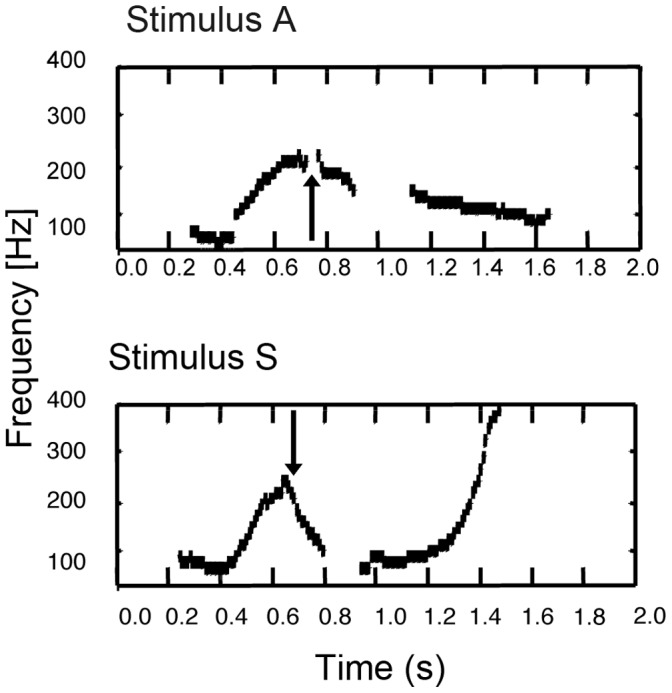
The typical pitch contours of the sentence “so’H desu ka” (Maekawa & Kitagawa (2000), partly corrected by the authors). The time axis is in seconds, and the zero point corresponds to the beginning of/s/. Arrows denote the timing of accentual fall. Sentences were spoken by speaker ST.

The finding of the present study is quite interesting, because, as shown in Figure 2, the beginning parts of the two phrases share similarities in increasing pitch. Conversely, the latter parts of the two phrases differ greatly. The “suspicion” prosody has increasing pitch but the “admiration” prosody does not. In fact, Japanese people discriminate between prosodic contours by listening to phrase-final pitch movement [13], [16]. Thus, it seems that although both humans and birds can discriminate between the prosodic features of human language, they focus on different cues in the prosody.

The role of auditory cues that were used was not clarified in the present study. There are many acoustic differences between speech stimuli, and it would be necessary to examine whether birds did not attend to some other speech cue. Differences in the magnitude of phrase-initial pitch rise or timing of accentual fall may be the cue. In addition, segmental features, such as vowel formant frequency, also change according to paralinguistic information [55]. These differences in suprasegmental and segmental features may be used as discriminative cues. Further investigation is required to resolve this issue. It would have been more reliable to use synthesized speech stimuli that controlled the segmental features of the stimuli, such as vowel formant frequency and/or a higher number of exemplars that share a given feature, and test them with a different set of new exemplars.

The results of Experiment 2 demonstrated that when the birds trained on sentence discrimination were tested on the same sentence with a different prosody (admiration), they were unable to discriminate between the two stimuli. These results suggested that the sparrows perceived the sentences with different prosody as different stimuli. As described in the introduction, bird songs share several aspects with human language. However, songbirds are not at a prelinguistic stage of human language, because human language and bird songs have evolved through different routes. Their neural system for conspecific perception is different from that of humans. Neurons in the nucleus robustus of the arcopallium (RA) responded selectively to the individual’s own song [56] and those in the Higher Vocal Center (HVC) in the nidopallium also showed preference for their own song [57]. Budgerigars, which are not passerine but have well-developed voco-auditory learning system, have an anatomically different system from that of passerine birds [58]. In addition to avian species, several other species, such as dolphins [59], elephants [60], and bats [61], show sophisticated auditory abilities. Thus, specialization of voco-auditory learning has evolved independently in different lines of evolution, which have evolved different neural mechanisms for a similar function. As demonstrated in the present experiment, the auditory system specialized for discrimination of conspecific song in Java sparrows can also be used for different problems.

In summary, the present study has demonstrated prosodic discrimination ability in Java sparrows. Furthermore, the sparrows attended to differences in prosody rather than the content of the sentence even though they could discriminate between differences in the sentence after training. Moreover, unlike Japanese speakers, who focus on the end of an utterance, Java sparrows seem to use the first part of an utterance as the discrimination cue. The present results suggest that the discrimination of prosody is a predominant ability not only in human infants (at an ontogenetically early stage) but also in phylogenetically different species that use vocal communication.

## Materials and Methods

### Experiment 1: Prosody Discrimination

#### Subjects

Five adult male Java sparrows (*Padda oryzivora*), kept at approximately 90% of their free-feeding weight, were used in Experiment 1. All birds were experimentally naive. The experiment reported here was conducted with the approval of the ethics committee of Keio University, Faculty of Literature.

#### Apparatus

**Figure 3:**
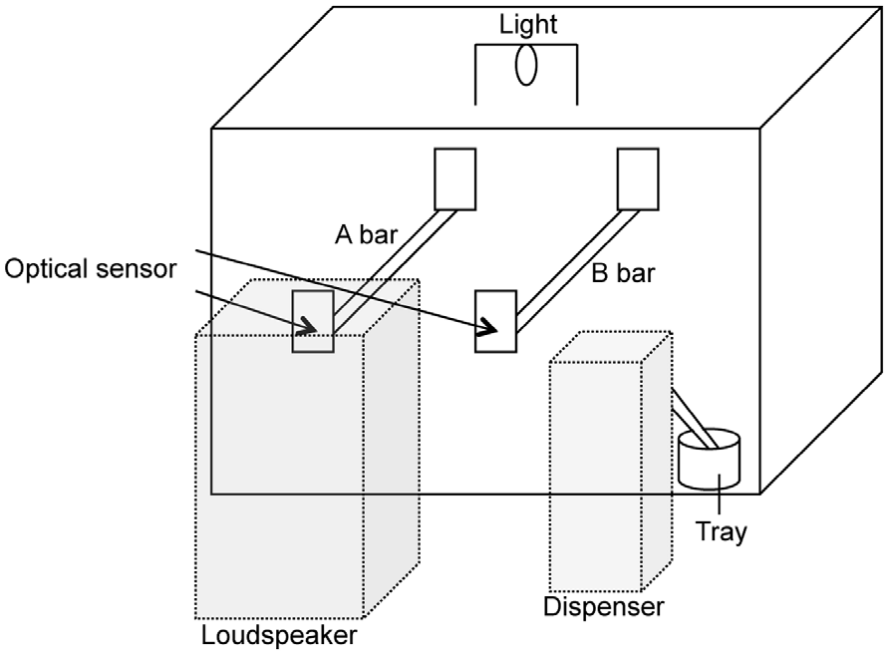
A diagram of the apparatus.

The experimental chamber was a cage (15×30×20 cm) with two perches (Figure 3). One was a ready perch (A bar), and the other, a response perch (B bar). A photo-sensor (OMRON, E3V-R2C43S) attached to each perch detected the position of the bird. A tray connected to a dispenser (Okubo Instruments, Tokyo) was placed in front of the B bar. The dispenser was designed to drop a few grains of millet onto the tray. The chamber was placed in a sound-insulated box (37×62×59 cm). A computer (Macintosh, Quadra 840 AV) placed outside the box controlled the experiment. A loudspeaker was connected to the computer and placed in front of the A bar presented in the stimuli. The cage had a small light on the ceiling.

#### Stimuli

A Japanese sentence, “so’H desu ka” (“ Is that so?” in Japanese), was used as the training stimulus. It was identical to the one previously described [55]. “Desu” is the polite form of the copula, and “ka” is a particle used at the end of interrogative sentences. The symbol “H” indicates the second element of a long vowel, and the apostrophe indicates the location of the lexical pitch accent on the noun before the copula. Speakers of standard Japanese read the sentence “so’H desu ka” to express six paralinguistic information types: admiration (“That’s great”), disappointment (“Forget it”), suspicion (“I don’t believe it”), indifference (“I’m not interested”), focused, and neutral. Speakers were trained until they could produce the intended message consistently.

A perception test was conducted to check the validity of these six paralinguistic stimuli (admiration, disappointment, suspicion, indifference, focused, and neutral) using adult human raters [55], [62]. The test indicated very high correct perception rates of all six paralinguistic information in Japanese participants (89% for admiration, 99% for disappointment, 98% for suspicion, 81% for indifference, 86%for neutral, and 59% for focused) compared to the chance level (17%). When the same perception test was conducted with American English-speaking participants who had learned Japanese to some extent, their correct perception rates were lower than that of Japanese participants (63% for admiration, 82% for disappointment, 79% for suspicion, 44% for indifference, 45%for neutral, and 45% for focused), but higher than the chance level (17%) [62]. In addition, when these six paralinguistic stimuli were judged by English-speaking participants who had not learned Japanese at all, their correct perception rates were lower than that of Japanese listeners and English listeners who had learned Japanese (24% for admiration, 46% for disappointment, 79% for suspicion, 39% for indifference, 29%for neutral, and 13% for focused), but still higher than the chance level (17%) except for “focused” [62]. Thus, these findings suggest that some prosodic cues to convey paralinguistic meanings are universal, but there are also language- or culturally-specific prosodic cues.

In the present study, we used only two messages: admiration (Stimulus A, Supporting Information Audio S1) and suspicion (Stimulus S, Supporting Information Audio S2), because they have similar durations and prominent differences in the pitch range at the end of the sentence (see Figure 2).

As shown in Figure 2, there are several differences between the two prosodic contours. Stimulus A is 0.3 s longer in duration. In Stimulus S, the pitch range becomes wider at the end of the phrase, the peak of the pitch is located at the second molar (de), and the decrement by accent nucleus is steeper. Furthermore, the pitch of Stimulus S increases at the end of the phrase, whereas that of Stimulus A decreases.

In Test 1, a novel text, “ana’ta desu ka” (“Is that you?”), uttered in admiration (Test Stimulus A) and suspicion (Test Stimulus S), was used as the test stimulus. The speaker of the test stimulus was the same as that during the training.

In Test 2, hybrids of the training stimuli were used. The phrase “so’H desu ka” was divided into two parts: “so’H desu” and “ka”. One hybrid had a prosodic contour beginning with admiration, followed by suspicion (Test Stimulus A+S), and the other had the reverse prosody (Test Stimulus S+A).

Each speech samples were analyzed using Praat [63], and mean pitch and pitch range of each sentence stimulus, and the beginning (“so’H desu” or “ana’ta desu”) and the latter part (“ka”) of the stimuli were calculated. Acoustical characteristics of the stimuli are reported in Table 1.

**Table 1:** Acoustic features of training and test stimuli.

	Total		The Beginning Part		The Latter Part	
	Mean Pitch (Hz)	Mean Pitch Range (Hz)	Mean Pitch (Hz)	Mean Pitch Range (Hz)	Mean Pitch (Hz)	Mean Pitch Range (Hz)
Training Stimuli						
Stimulus A	146.9	193.3	180.4	188.0	93.3	45.1
Stimulus S	174.3	267.0	184.9	203.4	162.8	267.0
Test Stimuli						
Test Stimulus A	154.9	217.4	203.2	217.4	92.9	48.3
Test Stimulus S	150.8	273.4	160.3	252.7	139.2	268.7

The mean pitch of the two training stimuli (Stimulus A and Stimulus S) were 146.9 Hz for Stimulus A and 174.3 Hz for Stimulus S. The mean pitch of the beginning parts of the two training stimuli (Stimulus A and Stimulus S) were very similar to each other (180.4 Hz for Stimulus A and 184.9 Hz for Stimulus S), whereas those of the latter parts (“ka”) were different in that higher pitch in Stimulus S (93.3 Hz for Stimulus A and 162.8 Hz for Stimulus S).

With respect to test stimuli (Test Stimulus A and Test Stimulus S), the similar pitch was observed in Test Stimulus A and Test Stimulus S (154.9 Hz for Stimulus A and 150.8 Hz for Stimulus S). The mean pitch of the beginning parts of Test Stimulus A was relatively higher than that of Test Stimulus S (203.2 Hz for Test Stimulus A and 160.3 Hz for Test Stimulus S). Similar to the training stimuli, the mean pitch of the latter parts of the stimuli (“ka”) were higher in Test Stimulus S, when compared to Test Stimulus A (92.9 Hz for Test Stimulus A and 139.2 Hz for Test Stimulus S).

All stimuli were presented at a measured intensity of approximately 60 dB.

#### Procedure

First, all birds were trained to stay more than 3 s on the A bar before moving to B bar. Then, the birds were divided into the Admiration Group, consisting of three birds, and the Suspicion Group, consisting of two birds. The stimuli were played through the speaker after a bird had stayed on the A bar for 3 s. In the Admiration Group, a move to the B bar within 3 s after the onset of the admiration prosody was positively reinforced (hit); responding to suspicion prosody (false alarm, FA) resulted in the light being turned off for 5 s. A hit or non-response for 3 s to the suspicion prosody (correct rejection, CR) started the next trial, whereas no response to the admiration stimuli (miss) or false alarm response resulted in a repetition of the same trial. These correction trials were repeated up to a maximum of five times. For the Suspicion Group, the procedure was the same, but the stimuli were reversed.

One session consisted of 40 trials in which the two stimuli were presented 20 times in random order. The training continued until the subjects attained a correct response rate (the sum of hit and CR trials divided by 40) above 80% on two successive sessions. Following the training sessions, the subjects received two different tests.

In Test1, novel text “ana’ta desu ka” (“Is that you?”), uttered in admiration and suspicion, was presented in the first test after discrimination training. If a bird had learned to differentiate between the two prosodic contours through training, the ability to discriminate should generalize the discrimination to the new phrases.

In Test 2, hybrids of the training stimuli were used. In Test 2-1, the birds were reinforced when the beginning part of the stimuli had a prosodic pattern associated with the reward in training. On the other hand, in Test 2-2, the responses to hybrid beginning part with the prosody not associated with food in the training were reinforced. Test 2-1 and Test 2-2 were presented in counterbalanced order between subjects. In addition, the birds were given each test twice and Test2-1 and Test 2-2 were presented alternately and not repeated twice in a row. In the test sessions, the birds had to respond within 5 s from the start of stimulus presentation. The limited hold was extended from 3 s as in training to 5 s in the tests, because we expected birds to hesitate before responding to novel stimuli. Following one test, the birds were retrained with the training stimuli until they again attained a correct response rate above 80% on two successive sessions to maintain their discrimination.

Each test consisted of one session of 40 trials, and the contingency of reinforcement was effective as the training session, but the correction procedure was not used. Reward during the tests might affect discriminative behavior of the subjects, but it is known that Java sparrows are very susceptible to extinction. Without food reward, they stopped the perching response completely. Since birds obtain reward for their correct responses during test, there is a possibility that the birds relearned discrimination of the test stimuli during the test. However, previous studies [37], [38] demonstrated that birds did not learn discrimination of the test stimuli through 40 trial-test session even when they were reinforced during the tests.

### Experiment 2: Sentence Discrimination

#### Subjects, apparatus, stimuli, and procedure

The specific methods and procedures were similar to those used in Experiment 1. Four experimentally naive male Java sparrows were trained to discriminate “so’H desu ka” and “ana’ta desu ka”. Both of them had prosody of suspicion. Two birds were reinforced with food rewards for responding to the phrase “so’H desu ka” (“so’H desu ka” Group) and the remaining two to the phrase “ana’ta desu ka” (“ana’ta desu ka” Group). The same text as the trained stimuli, but uttered in different prosody (admiration), was presented in the test after the discrimination training.

## Supporting Information

Audio S1 **Stimulus A.**
(WAV)

Audio S2 **Stimulus S.**
(WAV)
